# Utilizing High-Speed 3D DIC for Displacement and Strain Measurement of Rotating Components

**DOI:** 10.3390/ma18173974

**Published:** 2025-08-25

**Authors:** Kamil Pazur, Paweł Bogusz, Wiesław Krasoń

**Affiliations:** 1 Łukasiewicz Research Network, Institute of Aviation, Al. Krakowska 110/114, 02-256 Warsaw, Poland; 2 Institute of Mechanics and Computational Engineering, Faculty of Mechanical Engineering, Military University of Technology, Gen. Sylwestra Kaliskiego 2 Street, 00-908 Warsaw, Poland; pawel.bogusz@wat.edu.pl (P.B.); wieslaw.krason@wat.edu.pl (W.K.)

**Keywords:** digital image correlation, strain gauge, propeller deformation, measurement, rotational motion analysis, error analysis, noise reduction methodology, material characterization

## Abstract

This study explores the effectiveness of 3D Digital Image Correlation (DIC) for measuring displacement and strain of a propeller undergoing angular motion. Traditional methods, such as strain gauges, face limitations including physical interference, technical difficulties in sensor connections, and restricted measurement points, leading to inaccuracies in capturing true conditions. To overcome these challenges, this research utilizes non-contact 3D DIC technology, enabling measurement of surface displacements and deformations without interfering with the tested component. Experiments were conducted using the model aircraft propellers mounted on a custom-built test stand for partial angular motion. The 1 Mpx high-speed cameras captured strain and displacement data across the propeller blades during motion. The DIC strain measurements were then compared to strain gauge data to evaluate their accuracy and reliability. The results demonstrate that 3D DIC enables precise displacement measurements, while strain measurements are subject to certain limitations. Displacement measurements were achieved with a noise level of ±10 μm, while strain measurement noise ranged from 26 to 174 µm/m depending on direction. Strain gauge measurements were also performed for verification of the DIC measurements and calibration of the filtering procedure. Two types of non-metallic materials were used in the study: Nylon LGF60 PA6 for the propeller and 3D-printed PC ABS for the cantilever beam used in strain measurement validation. This study underscores the potential of DIC for monitoring rotating components, with a particular focus on measuring strains that are often overlooked in publications addressing similar topics. Additionally, it focuses on comparing DIC strain measurements with strain gauge data on rotating components, addressing a critical gap in existing literature, as strain measurement in rotating structures remains underexplored in current research.

## 1. Introduction

Studying the mechanical behavior of rotating components, such as rotor blades or turbine elements, presents unique challenges due to their dynamic environment [1,2]. Traditional measurement techniques, such as resistance strain gauges and optical methods, require complex signal transfer mechanisms like slip rings or telemetry systems to bridge the gap between the rotating and stationary parts of the acquisition system. It is also problematic due to the need to route and secure wires through the rotating shaft of the test object [3,4,5,6]. Contact measurement methods like strain gauges or optical fiber Bragg grating encounter numerous limitations that can substantially affect the reliability and accuracy of the results. The researchers of the study titled “Dynamic Measurement of Strain and Shape on a Rotating Helicopter Rotor Blade Using Optical Fibre Sensors” [6] encountered a problem with the accuracy of Fiber Bragg Grating (FBG) strain measurements. Amplitudes measured depended heavily on the placement of sensors relative to the structure’s neutral axes. This meant that identifying all modes required carefully optimizing the sensor layout, which in turn required a thorough understanding of the blade’s structure.

One major issue with contact methods is their interference with the element being measured. Introducing physical contact with a rotating element can alter its dynamics, leading to unwanted changes in behavior, introducing an error. Moreover, mounting sensors on rotating parts can be technically difficult or even impossible, especially for small-sized elements or those operating at high speeds. Another problem is the limited number of measurement points offered by contact methods. Strain gauges also have a limited measurement range, typically around 5%, beyond which they become detached and damaged. This limited measurement capability does not allow for a comprehensive understanding of phenomena occurring on the surface of rotating components, which sometimes are crucial for ensuring their designed proper functions [1].

The solution to these issues appears to be the use of non-contact technologies, such as 3D Digital Image Correlation (DIC), which offers the ability to measure surface displacements and deformations. This technology utilizes image processing and photogrammetry algorithms, which enable tracking and analysis of changes on the surface of elements without the need for direct contact.

Developed in 1988, DIC became commercially used in the early 2000s, evolving from an academic curiosity to an industry-standard solution for structural displacement and strain analysis. Bing Pan, in his review of the historical development, highlights that “In the experimental mechanics community, DIC has been considered as a revolutionary advance; the most important advance since the strain gauge. It will continue to be the most practical and powerful deformation measuring tool for the foreseeable future” [7].

The development of DIC has advanced to dynamic measurements; exemplarily, it has been used in tensile split Hopkinson bar experiments, where it provides full-field strain and strain rate data. By utilizing high-speed cameras, the DIC allowed for detailed analysis of strain distribution, including necking phenomena, offering insights that traditional methods could not achieve [8].

A recent study by Jonsson and Kajberg [9] demonstrated the use of 3D Digital Image Correlation in dynamic axial crash testing of metal crash boxes, highlighting the growing application of DIC in high-speed experimental mechanics. Their results showed that 3D-DIC can effectively capture full-field strain, deformation, and crack propagation during dynamic loading, supporting its use in crashworthiness evaluation and impact-related studies.

The 3D DIC is also being successfully applied in high-speed bird strike testing. L. Barrière demonstrated the use of stereo-DIC with high-speed cameras to measure full-field displacement and strain on aircraft shielding panels during impact, highlighting its effectiveness in evaluating deformation across large areas under dynamic loading [10].

The widespread adoption of Digital Image Correlation (DIC) technology has been driven by significant advancements in high-speed camera hardware and the development of more efficient and accurate correlation algorithms [11]. As noted by Philip Reu from Sandia National Laboratories [12], these improvements have played a key role in transforming DIC from a laboratory method into a practical tool for industrial applications.

The introduction of 3D DIC to the measurement of rotating components opens up new possibilities for diagnosing and monitoring their condition. For instance, a recent study by P. Sousa [13] applied 3D DIC to monitor the deformation of a rotating RC helicopter blade under dynamic loading at 680 rpm. The setup utilized high-speed cameras triggered by a laser-based system, enabling precise acquisition of displacement data. The study demonstrated that using Digital Image Correlation to monitor displacements of a blade rotating at 680 rpm is feasible; however, it does not include an analysis of strain.

Another article by Z. Su [14] presents a stroboscopic stereo-DIC system specifically designed to measure the full-field 3D dynamic displacement of underwater rotating marine propeller blades, addressing key challenges such as light refraction, camera calibration, and image clarity. However, the results focus on displacement fields and do not cover strain measurement.

This study investigates the applicability of Digital Image Correlation (DIC) for strain analysis of a partially rotating propeller and evaluates its accuracy by comparing the results with strain gauge measurements. The literature review revealed that strain measurements of rotating components are often overlooked, whereas this study focuses specifically on their analysis using Digital Image Correlation. The analysis conducted in this work is both qualitative and quantitative, providing an evaluation of DIC performance. Furthermore, the research aims to validate the effectiveness of the DIC method in analyzing propeller displacement. A general methodology for the static validation of DIC parameters such as subset size, subset step, and filtering is also outlined, based on a reference bending beam test. This work also aims to contribute to the development of a practical testing procedure for rotating elements, with a particular focus on propellers.

## 2. Digital Image Correlation Validation

### 2.1. Algorithms Behind the Curtain

Three-Dimensional Digital Image Correlation is a non-contact method for measuring deformations on the surfaces of objects using optical techniques. This method incorporates a stereo vision setup involving two cameras that capture images from different angles. Cameras record the speckled stochastic patterns on the object’s surface, which change as the object deforms.

The process begins by capturing baseline images of the object at rest. When a load is applied, the object deforms, and subsequent images are taken to capture these changes. The key computational challenge is to match the speckle patterns between the deformed and baseline images to compute the full-field displacement and strain on the object’s surface. This can be effectively achieved using the 3D Digital Image Correlation (3D DIC) method in conjunction with photogrammetry, which enables precise tracking and analysis of surface deformation.

In the field of DIC, a variety of algorithms are employed to analyze the deformation and displacement of materials under stress. Notable among these are the Lucas-Kanade algorithm [15], which is foundational for tracking motion between images; the Gauss-Newton algorithm, known for its efficacy in minimizing the sum of squared differences in deformations; and its variants, such as the Inverse Compositional Gauss–Newton and Forward Compositional Gauss–Newton [16,17], which enhance computational efficiency and convergence. Additionally, the Newton-Raphson algorithm, traditionally used for finding roots of a real-valued function, is adapted in some DIC applications to iteratively refine the solution of displacement fields.

Despite the critical role these algorithms play in ensuring precise and reliable analysis, providers of DIC software often do not disclose which specific algorithms are implemented within their products, nor the exact methods used for calculating strain and displacement. This limited clarity regarding the mathematical basis behind the results may present challenges for users when attempting to verify the capabilities and limitations of the software in both research and industrial applications. This can be particularly important in the study of dynamic, fast-changing processes. Knowing and understanding the underlying algorithms is crucial for ensuring consistent and reliable uncertainty quantification in DIC measurements. At the IEEE IST 2022 conference, Phillip L. Reu, the president of the International Digital Image Correlation Society (iDICs), highlighted that standardization and certification of the DIC method remain among the most urgent challenges, as they are crucial for uncertainty tracing and improving measurement consistency [12]. This study focuses on comparing DIC strain measurements with strain gauge data on rotating components, addressing a critical gap in existing literature, as strain measurement in rotating structures remains underexplored in current research.

### 2.2. Three-Dimensional DIC Uncertainty Estimation Approaches

The field of 3D DIC currently faces significant challenges in estimating measurement uncertainties. Traditional methods for uncertainty quantification in DIC are underdeveloped, primarily due to the complexity and nonlinearity inherent in the measurement processes involved [18].

**Figure 1:**
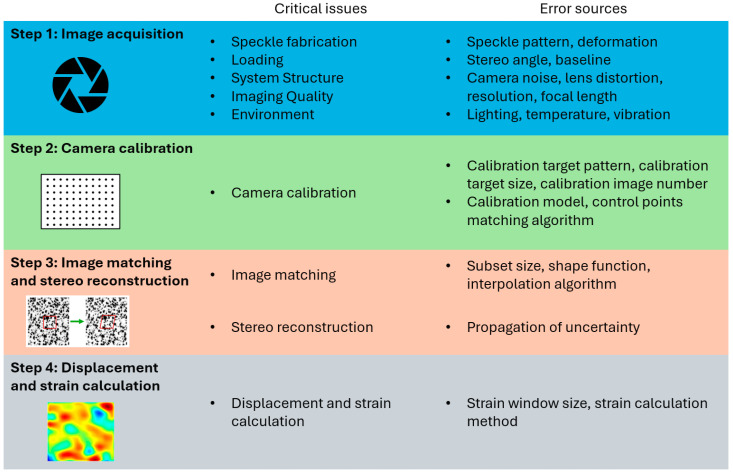
Schematic diagram of 3D DIC measurement steps and the sources of uncertainty for each DIC step [19].

Estimation of measurement uncertainties for Digital Image Correlation 3D requires analysis of measurement error sources at each of the four steps of measurement. These steps are image acquisition, camera calibration, image matching, and stereo reconstruction, all of which introduce potential sources of errors that are difficult to isolate and quantify—Figure 1 [19]. The propagation of these errors through each stage of the measurement process makes it extremely challenging to ascertain the true uncertainty of the final measurement results. Factors such as the quality of the camera calibration, the precision of image matching, and the integrity of the stereo reconstruction all play critical roles in the accuracy and reliability of DIC measurements. The limited information available about the functioning of algorithms in software used for DIC video analysis also complicates the estimation of measurement uncertainty [18].

In response to these challenges, the authors of the article [19] propose a novel approach that integrates Monte Carlo simulation with theoretical analyses of camera calibration and DIC random errors. Their method focuses on simplifying the uncertainty estimation process without the need for additional experimental procedures. By theoretically estimating the errors introduced during camera calibration and image matching and then using Monte Carlo methods to simulate these errors’ propagation through the DIC measurement chain, this approach allows for a more practical and computationally efficient estimation of measurement uncertainty [19].

In the field of engineering and technology, standards play a crucial role in the consistency, reliability, and quality of processes. In the field of 3D scanners and digital image correlation methods, there are VDI/VDE 2634 Part 1 and VDI/VDE 2626 standards, which are well-recognized within the industry [20]. These standards have been developed by organizations: the Association of German Engineers (VDI) and the Association for Electrical, Electronic, and Information Technologies (VDE).

The VDI/VDE 2626 guideline outlines how to perform a procedure that verifies the system’s capability to accurately track and measure displacement and strain changes. These tests are critical for confirming that DIC systems provide reliable and repeatable measurements, which are essential for structural analysis, material testing, and quality control [21]. However, the practical application of VDI/VDE 2626 to custom or mobile DIC setups presents significant challenges. For instance, each time a custom DIC system (e.g., each setup with a high-speed camera) is configured, the VDI/VDE 2626 procedure would need to be repeated to estimate uncertainties for the new setup. This is both time-consuming and impractical in many testing environments, especially where rapid setup changes are necessary.

For a custom camera DIC system, uncertainty estimation becomes a more complex task, as any change in the lens angle or depth of field will affect the resulting measurement uncertainties. This dynamic recalibration is crucial for applications involving large structures or in-field testing, where environmental and setup conditions can vary significantly. The VDI/VDE 2626 standard is practical for fixed DIC setups. One common approach for quantifying variance error is to use images of not loaded test object and quantify uncertainty basing on measurement noise. This solution is mentioned in the Good Practices Guide for Digital Image Correlation by the International Digital Image Correlation Society (iDICs) [22]. This approach allows us to anticipate what variance error can be achieved on a particular camera setup before proceeding with actual testing. This approach is more convenient for adapting DIC technology to dynamic and less controlled environments, where setup conditions may frequently change [23,24].

### 2.3. Strain Measurement Validation

For the purpose of validating DIC strain measurements, a validation setup was prepared along with a validation procedure. Its purpose is to confirm the accuracy of strain measurements using DIC technology. The primary goal of the procedure is to optimize parameters such as subset size and filtering settings in the context of a specific camera setup.

In Digital Image Correlation (DIC), there is a critical trade-off between noise and bias error, influenced by the selection of processing parameters. Using larger subsets and applying a larger spatial filter, and consequently a larger Virtual Strain Gauge (VSG), effectively reduces measurement noise, resulting in smoother strain fields. However, these adjustments also introduce bias errors and reduce the spatial resolution of the strain measurements. Thus, optimizing DIC parameters requires balancing noise suppression with minimizing bias to achieve reliable and accurate strain measurements tailored to the specific application [22].

The VSG size, expressed in pixels, does not necessarily correspond to the same physical area on the specimen surface as that covered by a traditional strain gauge. While the VSG represents the local region used for strain calculation, its physical equivalence to the strain gauge area is only approximate and depends on the specific strain computation method used in the DIC software [23]. For this reason, a validation of the VSG size was carried out by adjusting it to match the strain measurements obtained from traditional strain gauges.

The procedure involves capturing images of a bent beam equipped with strain gauges in a predefined position using a 3D camera system. Subsequently, a comparative analysis between the DIC measurements and the strain gauge readings is conducted. Based on this comparison, the DIC parameters, such as subset size and filtering settings, can be defined to ensure that the resulting strain measurements align with those obtained through the strain gauge method.

**Figure 2:**
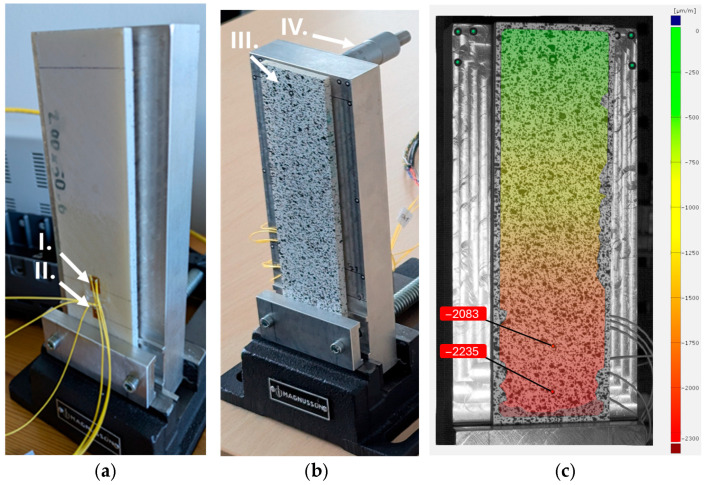
(**a**) Validation setup used for DIC strain measurement verification: I. Strain gauge 1, II. Strain gauge 2. (**b**) III. PC-ABS beam with speckled pattern applied. IV. Micrometer screw inducing the deflection of the beam. (**c**) Strain distribution map at a 9 mm displacement of the screw. Two marked VSGs correspond to strain gauges 1 and 2.

The validation setup for the Digital Image Correlation measurement consists of a PC-ABS beam, measuring 200 mm × 50 mm × 6 mm, which was fabricated using the FDM (Fused Deposition Modeling) method on a Zortrax M300 printer (Zortrax S.A., Olsztyn, Poland). The beam is subjected to controlled bending during the validation process. The beam is securely clamped in a cantilever-type mount, as shown in Figure 2a,b, allowing for controlled bending at the free end with an adjusting screw Figure 2c. In this setup, two strain gauges are mounted on one side of the beam to provide direct strain measurements during bending. The side visible in Figure 2a holds the strain gauges and serves as a reference for validating the strain data obtained from the DIC. The strain gauges (CEA-13-125UWA-120 by Micro-Measurements Raleigh, Wendell, NC, USA) are positioned along the central vertical line of the beam, with strain gauge 1 located 120 mm from the point of force application and strain gauge 2 positioned 140 mm from the force application point. Both strain gauge measurement channels measured with the NI 9237 module have been validated with a certified decade resistor. Virtual strain gauges (VSG) in the DIC analysis were placed at corresponding locations. On the opposite side of the beam, a random speckle pattern has been applied, essential for DIC analysis (Figure 2b).

The bending was applied using a screw-driven mechanism, allowing for a displacement range of 0 to 9 mm. The DIC setup includes two phantom VEO cameras with 1 MPx monochromatic sensors with 20 µm pixels, positioned at a stereo angle of 18.6 degrees. Nikon (Tokyo, Japan) Nikkor 50 mm lenses were used with an f/8 aperture setting and 100 µs exposure time. The scale factor achieved with this setup was around 0.2107 mm/px (measured at the left camera view on the speckled pattern plane). The strain measurements from both the strain gauges and the DIC system were recorded simultaneously to ensure direct comparability.

**Figure 3:**
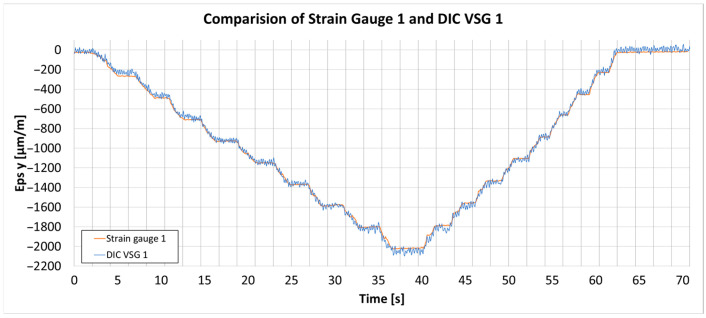
Comparison of the strain gauge 1 (orange) placed 120 mm from the bending screw and the corresponding DIC y strain (blue) measurement.

Figure 3 presents the graphs of the strain comparison between the DIC measurements and the strain gauge. In this setup, parameter adjustments were carefully made to balance the trade-off between noise and bias in the strain measurements. Parameters such as subset size and step, along with the temporal and spatial filters, were adjusted to find an optimal balance [25].

The final selection of these parameters was based on achieving a suitable compromise for this bending test. Initially, the subset size and subset step were chosen to ensure an acceptable level of input noise while maintaining a sufficient subset density to allow effective spatial filtering. Once the raw noise level was controlled, the spatial filter was gradually increased to reduce the measured strain values to match those obtained from strain gauges; a filter size of four was found optimal. Finally, a temporal filter of four was applied to further suppress measurement noise, with care taken not to over-smooth the data, as these parameters are intended for use in future dynamic testing of the propeller.

In the end, parameters were selected as follows: subset size 21 px, subset step 16 px, average spatial filter 4, and average temporal filter 4. The parameters optimized during this validation will be applied in further tests involving the actual propeller. The DIC system’s bias error in this setup remains below 4%, and the noise level is within ±30 µm/m, ensuring firm strain measurement.

### 2.4. Tensile Characterization of APC Propeller Blades Material

For the tests presented in this study, propellers manufactured by APC Propellers—Landing Products Inc. (1222 Harter Ave., Woodland, CA, USA) were used. To obtain accurate mechanical properties for subsequent finite element (FE) modeling, tensile tests were performed on the Complet LGF60-PA6 material, which was sourced from a propeller manufactured by APC Propellers [26]. The manufacturer’s datasheet reports a tensile modulus of ~21,900 MPa and tensile strength of ~243 MPa for this material. However, these nominal values (based on standard test specimens) may not reflect the actual behavior of the propeller blade. Therefore, a series of custom tensile tests was conducted on five dog-bone specimens (shaped according to ASTM D638 [27]/ISO 527 [28]) extracted directly from an APC 16 × 4E propellers to determine reliable material parameters. Such measured parameters (e.g., tensile modulus and Poisson’s ratio).

**Figure 4:**
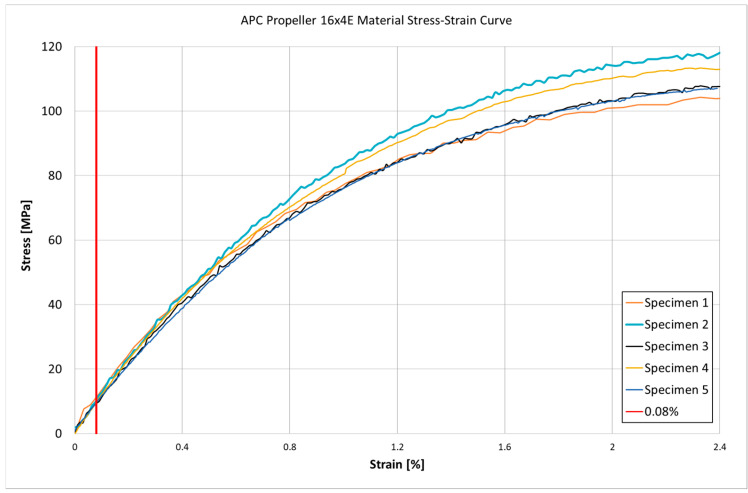
Tensile stress–strain curve for a specimens extracted directly from an APC 16 × 4E propeller.

The tensile tests were carried out using an Instron 8802 servohydraulic universal testing machine. A constant crosshead speed of 2 mm/min was used, corresponding to a quasi-static strain rate in line with ASTM D638/ISO 527 standard recommendations for polymer composites. The load was measured with the machine’s calibrated load cell, and crosshead displacement was recorded throughout each test. Strain was measured using two methods for improved accuracy. First, a contact axial extensometer (attached to the specimen gauge length) recorded the direct elongation of the sample. Second, a Digital Image Correlation (DIC) system (Aramis 3D, GOM GmbH, Braunschweig, Germany) was employed to capture full-field strain. Figure 4 and Table 1 present the results of the tensile tests, including the stress–strain curves and the measured material parameters such as tensile modulus, tensile strength, and Poisson’s ratio. Tensile modulus was calculated as the slope of the stress–strain curve in the initial linear range from 0% to 0.03% strain, following the procedure for chord modulus determination given in ISO 527. Test conditions for molding and extrusion plastics.” Moreover, during all five tests the recorded strain never exceeded 0.08%. This level, indicated by a red vertical marker in Figure 4, confirms that the material response discussed here falls entirely within the elastic region.

The experimental tensile tests conducted on five dog-bone specimens extracted directly from an APC 16 × 4E propeller made of LGF60-PA6 revealed significant deviations from the nominal material properties reported by the manufacturer. While the datasheet lists a tensile modulus of approximately 21,900 MPa and a tensile strength of 243 MPa, the values obtained from the actual propeller material were substantially lower. The measured tensile modulus ranged between 10,259 MPa and 10,794 MPa, while the tensile strength varied between 105 MPa and 119 MPa. These discrepancies emphasize the necessity of performing independent mechanical characterization on the final component, as processed parts may exhibit significantly different properties from datasheet values. The obtained experimental data will serve as a more realistic basis for subsequent finite element modeling of the propeller under operational loads.

## 3. Test Setup of Partially Rotating Propeller

**Figure 5:**
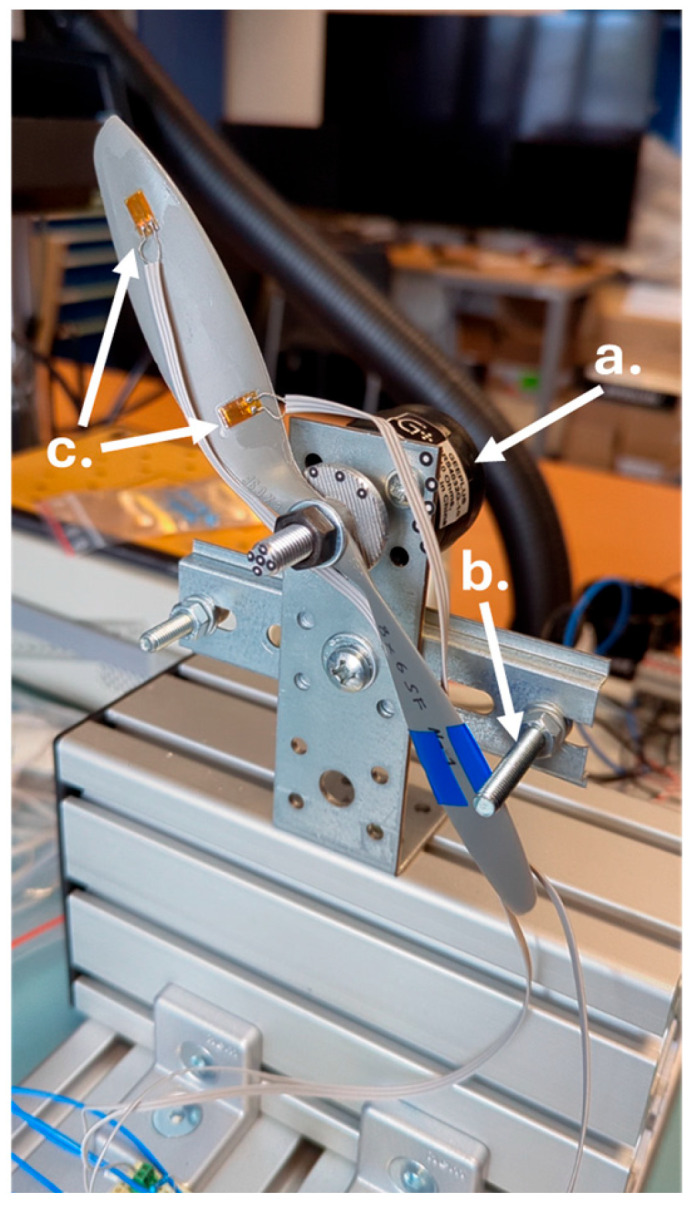
Test stand based on rotary solenoid: a. rotary solenoid gee plus; b. motion limiter; c. strain gauges.

**Figure 6:**
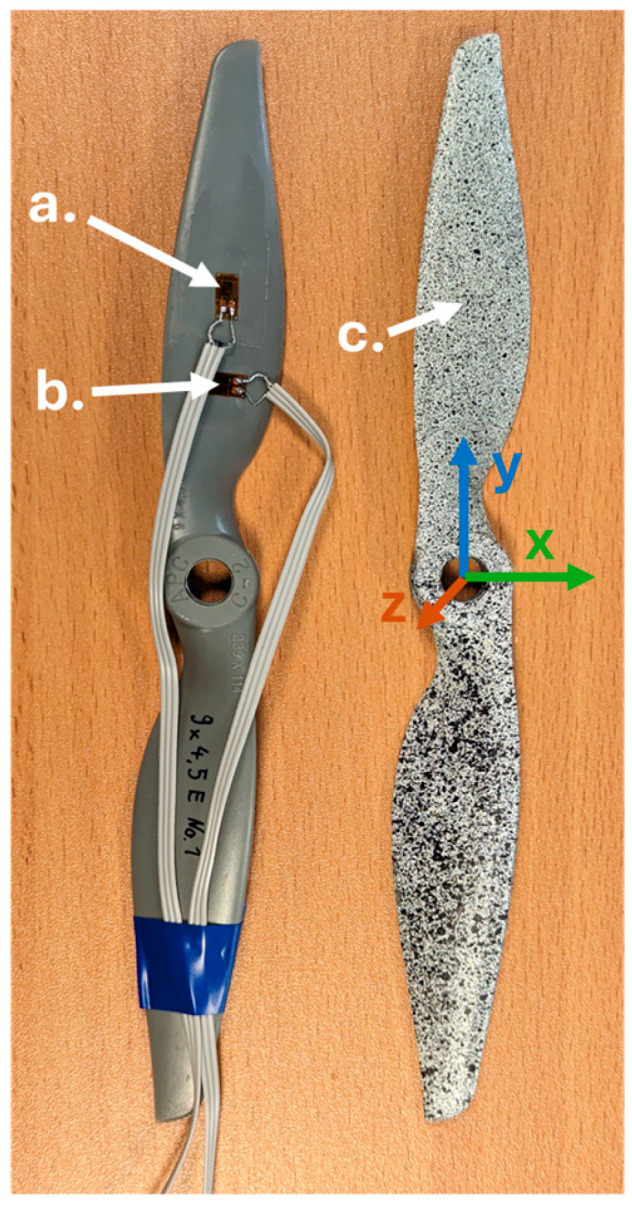
Propellers prepared for strain gauge and DIC test: a. *y*-axis strain gauge b. *x*-axis strain gauge c. propeller with stochastic pattern applied. On the right, the adopted propeller coordinate system is presented.

For the purpose of testing the capabilities of the DIC technique in studying the deformations of rotating elements, a special test stand was created. The test stand was dedicated to the study of model aircraft propellers, Figure 5 and Figure 6, where the angular motion is limited to 60 degrees and is performed by a fast rotary solenoid—Figure 5. The test stand has been designed to eliminate the need for additional strain gauge signal transmission systems, such as slip rings or telemetry.

The research was conducted using the model aircraft propellers manufactured by APC Propellers. Two specific propellers have been selected for the experiments: APC 8 × 6SF and APC 9 × 4.5E. The first number before x corresponds to the propeller’s diameter in inches, and the second number represents the propeller’s pitch in inches. Two high-speed cameras have been used in the experiment to capture the movements of the tested propellers, the Phantom VEO 710 and VEO 410 manufactured by Vision Research, Inc., Wayne, NJ, USA. Both cameras have the same 1 Mpx sensor, but the VEO 410 has lower pixel/second bandwidth, which limits the maximum recording frequency of the system—Table 2. This limitation arises from the requirement that, in a 3D DIC system, both cameras must capture frames simultaneously.

The study was conducted to assess the DIC method’s effectiveness in measuring partially rotating elements while also determining the precision boundaries and minimum detectable values of displacements and strains of the 1 Mpx high-speed cameras’ 3D DIC system. In the next step, the deformation measurements made with the DIC method will be compared with strain gauge measurements. On the designed test stand, the propeller performs a rapid partial angular motion, and then the lower part of the propeller strikes a mechanical limiter. During the test, three phases can be distinguished: the idle phase, the acceleration phase, and the impact phase. The maximum angular velocity before hitting the limiter was 1182 rpm for the APC 8 × 6SF and 720 rpm for the APC 9 × 4.5E propeller (calculated based on the kinematic analysis of points on the propeller using DIC). For the analysis, model aircraft propellers from APC were used. All the propellers are made from Complet LGF60-PA66 [26], which is nylon reinforced with 60% glass fiber. A stochastic pattern in the form of random black dots on a white background was applied to the propellers. The coordinate system was adopted as shown in Figure 6, where the *y-axis* corresponds to the longitudinal direction and the *x-axis* corresponds to the transverse direction of the propeller.

For each propeller, two DIC recordings were performed: Test Runs 1 and 2 for the APC 9 × 4.5E propeller and Test Runs 1 and 2 for the 8 × 6SF propeller. Table 2 presents the parameters of recording with high-speed cameras along with calibration parameters. It was necessary to use a low exposure time to ensure that the image of the rapidly moving propeller was not blurred by motion. Exposure time was calculated according to the maximal expected linear velocity (14.14 m/s) of the propeller tip. To minimize the motion blur, the rule was adopted that in the given frame, the displacement of the propeller tip should not exceed 1 pixel during the exposure time. Therefore, the 10 µs exposure time was required. In 10 µs, the tip of a 228.6 mm (9 inch) diameter propeller rotating at 1182 rpm will travel approximately 0.637 pixels in an image with a scale factor of 0.2219 mm/px.

After conducting tests using 3D DIC, subsequent experiments were carried out using propellers equipped with the strain gauges, while maintaining all other test parameters constant. The strain gauges were placed along the longitudinal and transverse axes of the propellers—Figure 6. These gauges were connected to measuring equipment that recorded changes in resistance within the strain gauges during the angular movement of the propellers. Data acquisition was conducted using an NI (NI, an Emerson company; 11,500 North MoPac Expressway, Austin, TX, USA) cDAQ controller with an NI9237 strain gauge bridge measurement card. The measurement card’s inputs supported both full and half-bridge configurations, necessitating the use of external bridge completion with resistors matched to the resistance of the selected strain gauges (Micro-Measurements CEA-06-125UN-120), specifically 120 Ω with a tolerance of 0.25%. The acquisition was carried out at a sampling rate of 30,000 samples per second. Before the series of tests, the measurement system was verified using a calibrated decade resistor. The setup of strain gauges on the propellers and their parameters were recorded for each propeller. The strain gauges were precisely positioned relative to the axis of rotation using a caliper, and their locations were documented for subsequent strain analysis using the DIC method. Precise positions of the strain gauges are essential for aligning the corresponding measurement points on the deformation map in the 3D DIC method.

## 4. Analysis and Results

Measurement data from the 3D DIC system were obtained using GOM Correlate 2018 software (GOM Correlate, a ZEISS company; Carl-Zeiss-Strasse 22, Goslar, Germany) [25]. The displacement and strain maps of propeller blades during tests on a partial angular motion test stand were analyzed. After conducting the studies using DIC, corresponding tests were performed using strain gauge measurements at the same configuration.

**Figure 7:**
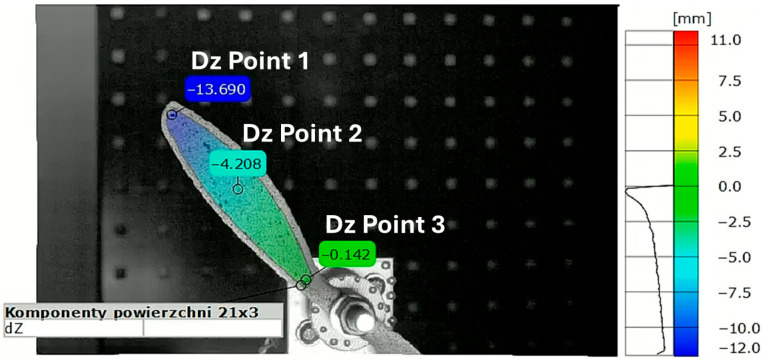
Test Run 2. Z displacement map of APC 8 × 6SF at 35.8 ms with displacement measurement point marked.

**Figure 8:**
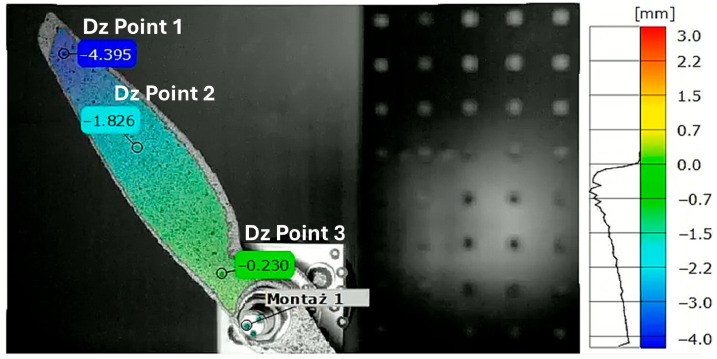
Test Run 2. Z displacement map of APC 9 × 4.5E at 10.4 ms with displacement measurement point marked.

**Figure 9:**
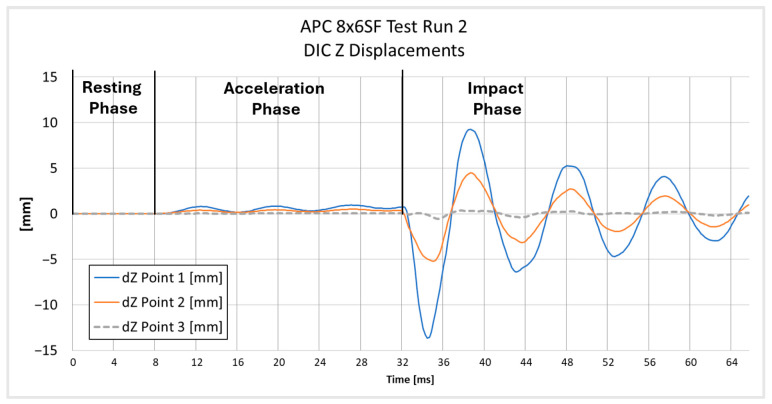
Component displacement measured with DIC at three points on a propeller during Test Run 2 of APC 8 *×* 6SF.

**Figure 10:**
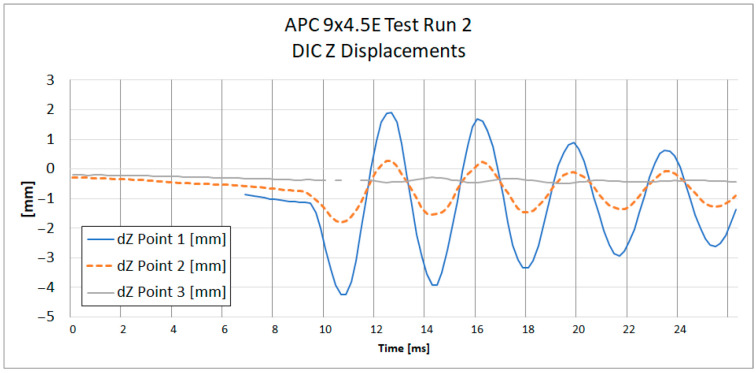
Z-direction displacement measured with DIC at three points on a propeller during Test Run 2 of APC 9 *×* 4.5E.

The displacements were measured along the *Z*-axis, i.e., the axis perpendicular to the plane of the camera sensor (depth axis). The local coordinate system was linked to markers placed at the center of the rotor. The local coordinate system rotated together with the propeller motion, ensuring that the strain measurements remained consistent with the orientation of the strain gauges mounted on the propeller. The analyzed points were located first near the propeller tip, second in the middle of the blade length, and third near the hub. Figure 7 and Figure 8 present the positions of each point on both tested propellers. The graphs presented in Figure 9 and Figure 10 show the *Z*-axis displacements of the corresponding three points.

The measurement uncertainty was estimated based on the measurement noise recorded in the propeller resting phase. The estimated uncertainty in the measurements is about ±10 µm, which is considerably smaller than the observed displacement amplitudes. At the beginning of the graph for the APC 9 × 4.5E Z Displacements on Figure 10, it is noted that tracking for Point 1 was lost due to the point leaving the frame. This loss of tracking results in the absence of data for Point 1 during the initial period of the measurement. In Figure 9, all three phases of motion are clearly visible: the resting phase (0–9 ms), the acceleration phase (9–32.1 ms), and the impact phase (32.1–64 ms). A small level of uncertainty affirms the reliability of the DIC displacement measurement.

In the subsequent phase of the analysis, strains measured using digital image correlation were compared with those measured using strain gauges. The strain gauges were placed along the longitudinal *y*-axis and the transverse *x*-axis of the propellers.

**Figure 11:**
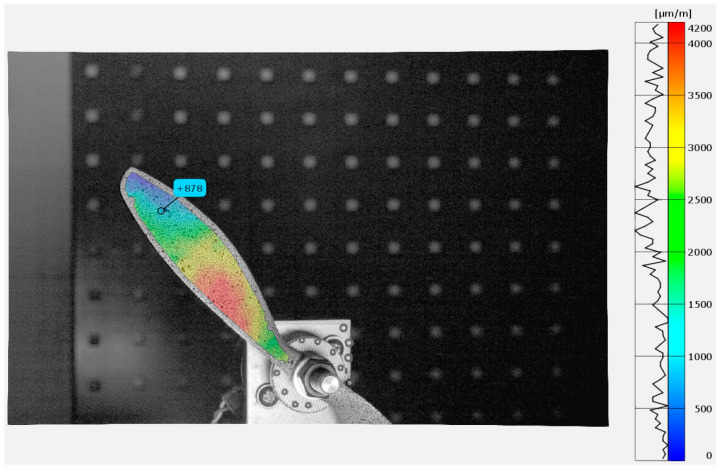
Y component strain map of APC 8 *×* 6SF during Test Run 2 at 35.8 ms.

**Figure 12:**
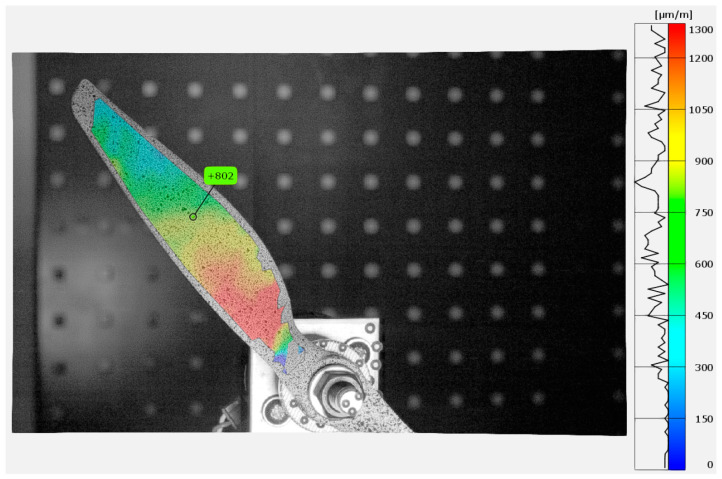
Y component strain map of APC 9 *×* 4.5E during Test Run 2 at 10.4 ms.

In Figure 6 in the DIC analysis, VSGs were positioned at locations corresponding to the strain gauges. GOM Correlate 2018 software was used for the analysis. Figure 11 and Figure 12 show the strain distribution along the y direction (longitudinal axis) during the propeller’s impact into the limiter at the highest strain amplitude. Additionally, there are marked positions of simulated strain gauges that have been adopted in DIC for the data comparison. The same parameters used during the validation on the validation setup were applied for the propeller test analysis. These parameters included a subset size of 21 px, a step size of 16 px, a temporal filter of 4, and a spatial filter of 4.

**Figure 13:**
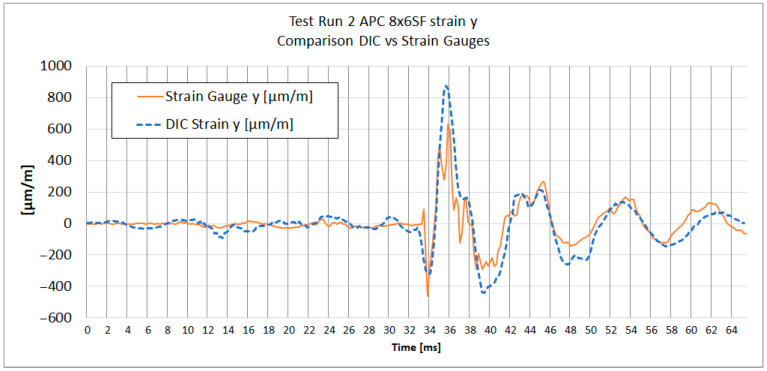
Test Run 2. Y-direction strain measurement of APC 8 × 6SF measured at the same point with DIC and a strain gauge.

**Figure 14:**
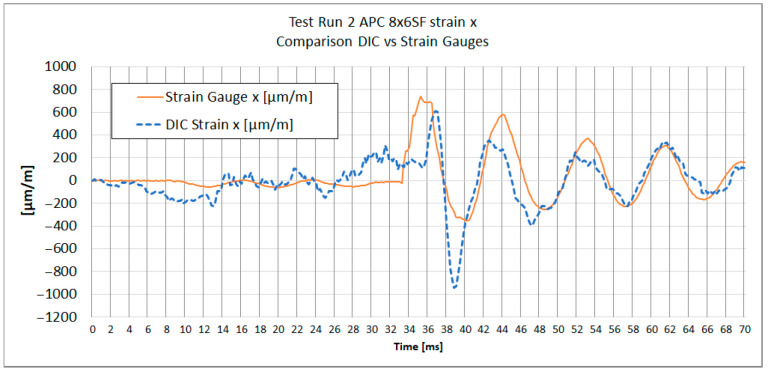
Test Run 2. X-direction strain measurement of APC 8 *×* 6SF measured at the same point with DIC and a strain gauge.

**Figure 15:**
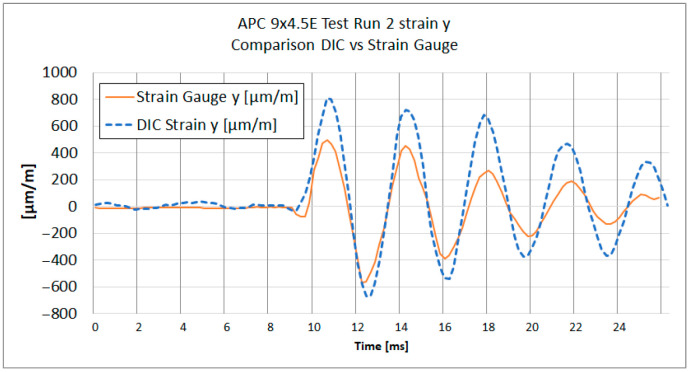
Test Run 2. Y-direction strain measurement of APC 9 × 4.5E measured at the same point with DIC and a strain gauge.

**Figure 16:**
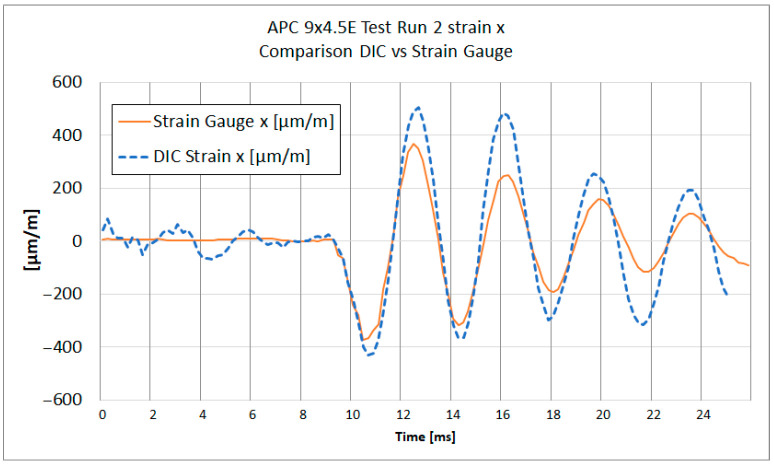
Test Run 2. X-direction strain measurement of APC 9 *×* 4.5E measured at the same point with DIC and a strain gauge.

Figure 13, Figure 14, Figure 15 and Figure 16 show the strain results of test run 2 obtained from both methods on the x and y axes for both tested propellers. The graphs from test run 1 correspond closely to the results from test run 2. For clarity and brevity of the article, only the graphs from test run 2 are presented.

The series of graphs presents a comparative analysis of strain measurements using both strain gauges and Digital Image Correlation (DIC) across two different axes (x and y) and for two different propeller models (APC 8 × 6SF and APC 9 × 4.5E).

In each graph, the DIC strain measurements show a broader range of values compared to the strain gauge readings. Strain gauges measure strain at specific discrete points where they are attached, averaging strain over the area of attachment. DIC analysis does the same; therefore, spatial and temporal filtering need to be carefully managed to obtain values as close to real as possible.

Both curves exhibit similar strain patterns over time, suggesting that both methods are consistent in detecting general changes in material strain. However, there are noticeable differences in the amplitude of measurements between the strain gauges and DIC. The curve representing DIC generally shows higher strain values and more pronounced peaks and valleys compared to the strain gauge curve, which is caused by the differences in the sensitivity and measurement accuracy of each method.

During the acceleration phase, when the strain levels are below the measurement noise threshold, certain fluctuations in the strain data were observed. Small inertial strains visible in strain gauge data likely influenced the DIC measurement trend, causing fluctuations that fall near or below the system’s measurement capabilities. A similar effect was noted in publication [29], where for strain values below 0.05%, a notable spread in results between DIC and strain gauge measurements was evident.

It is worth noting that the alignment of the strain gauges along the appropriate axis was performed using a caliper and a square, while the axes in the DIC method were determined based on the image. The misalignment of the strain axes between these two methods could potentially be a source of errors.

The provided Table 3 summarizes the comparison between DIC and strain gauge measurements across test runs, propellers, and strain components (x and y). Key parameters include noise levels, peak strain values, peak-to-peak strain of impact, the impact peak rise time, and Pearson correlation coefficient measured for both εx and εy.

The peak strain values indicate a considerable divergence between the DIC and strain gauge measurements. It was observed that dynamic strain measurements obtained using DIC exhibit a tendency to overestimate strain values. It can be noticed on Figure 15 and Figure 16, where subsequent local maxima and minima of strain recorded by DIC are higher than the strains measured with strain gauges.

A notable difference in strain measurement noise between the x and y axes was identified in DIC measurements. For TP1 8 × 6SF, the noise level for εx was ±167 µm/m, compared to ±26 µm/m for εy—Table 3. This difference may be due to the reduced number of neighboring subsets in the transverse direction of the propeller, corresponding to Ɛx. For strain gauge measurements, the measurement noise for both directions and for both propellers ranged from ±2.8 to ±6.9 µm/m.

Regarding the impact on peak rise time, the DIC method generally demonstrates a longer time to reach peak strain compared to strain gauges, as observed at TP1 for the 8 × 6SF propeller. In this case, the DIC records 3.7 ms compared to the strain gauge’s 2.8 ms in the x-direction. This suggests that strain gauges, which have been sampled at 30 ks/s, have a faster response and better capture the immediacy of rapid deformation events. The DIC system, operating at an image frame rate of 5000 fps (six times slower), exhibits a slightly delayed response. This delay is likely attributed to the temporal filtering applied, which in this case was set to ±4 frames.

A comparison of strain graph profiles obtained using the DIC method and strain gauges reveals that all profiles are similar, with the exception of the x-direction strain measurement for the 8 × 6SF propeller (Figure 14). For TP2 8 × 6SF εx—Figure 14—a significant deviation is evident in the DIC results compared to those from strain gauges. This discrepancy is likely influenced by the positioning of the measurement point within the lower, narrow section of the propeller. In this region, an artifact was identified, characterized by an apparent accumulation of strain. This phenomenon is presumably due to the proximity to the edge of the stochastic pattern and the reduced number of subsets available in this area.

The Pearson correlation coefficient was calculated for all test runs to evaluate the similarity between strain measurements obtained using the DIC method and strain gauges. The analysis was performed separately for the x and y axes, starting from the moment of impact to the end of the recorded data. The results show a generally strong correlation between the two methods, particularly in the *y*-axis measurements, where coefficients range from 0.84 to 0.90. In the *x*-axis, high correlation was observed in three out of four cases (0.94, 0.79, and 0.90), while one case (TR2 8 × 6SF) showed a significantly lower value (−0.16), indicating a potential issue in the DIC measurement for that specific test. This deviation is likely related to previously discussed artifacts in the lower section of the propeller, where strain accumulation and reduced subset density were observed.

## 5. Summary and Conclusions

This research investigated the use of high-speed 3D Digital Image Correlation (DIC) for measuring displacement and strain on rotating components, focusing on model aircraft propellers made from glass-fiber-reinforced polyamide (LGF60-PA66). A custom test rig was developed to enable partial angular motion of the propeller while capturing strain data using high-speed cameras and strain gauges. To validate the DIC results, complementary strain gauge measurements were performed, allowing for direct comparison. The study focused on the rotating structural components made of non-metallic materials, selected for their ability to undergo sufficiently large strains required in experimental setups. For the tested APC propeller made of complete LGF60-PA66, the essential mechanical characteristics were determined to support further FEM analysis.

The experimental results demonstrated that DIC provides accurate displacement measurements, with a noise level of ±10 µm, confirming its effectiveness. However, strain measurements exhibited higher amplitudes and increased noise levels compared to strain gauges. The strain values measured by DIC tended to be overestimated. The higher noise levels observed in DIC strain measurements (±26 to ±174 μm/m) compared to strain gauge measurements (±2.8 to ±6.9 μm/m) result from the inherent characteristics of the DIC technique, particularly under dynamic testing conditions. In this study, the subset size and spatial filtering were carefully optimized to balance noise reduction with spatial resolution, ensuring practical applicability to rotating components. The measurements were conducted using 1 MPx high-speed cameras, which limited the achievable subset size, density, and filtering effectiveness. It is anticipated that the use of higher resolution and frame rate cameras in future studies will enable the application of larger and more densely distributed subsets, reducing the strain measurement noise of 3D DIC in dynamic testing scenarios. The estimated noise level of the DIC method should be taken into consideration when selecting a measurement technique for a specific phenomenon. The results indicate that DIC may not be suitable for capturing low-magnitude elastic strains that do not exceed a few hundred μm/m.

Based on the conducted research, the following conclusions were drawn:

- The study of rotor blades in angular motion demonstrated that displacement and strain measurements of rotating objects using 3D DIC with a setup involving 1 MP monochromatic high-speed cameras are feasible, though with certain limitations. These limitations primarily relate to the virtual strain gauge (VSG) size and subset density, as accurate strain analysis requires high-resolution images that enable the use of larger VSGs to reduce measurement noise. Additionally, a high frame rate is beneficial for applying temporal filtering. Therefore, the achievable accuracy is directly constrained by the specifications of the available camera system.
- Based on static comparative measurements of the bending beam, spatial and temporal filtering parameters were determined, allowing for a strain distribution assessment using the DIC method. Based on the conducted calibration, the random error and bias were reduced. The bias was found to be below 4%, and the noise level was within ±30 µm/m. The determined filtering parameters were applied in dynamic tests.
- The displacement measurement uncertainty, estimated at ±10 µm during the propeller resting phase, affirms the reliability of DIC for capturing Z displacements. This is a relatively small value compared to the measured values, indicating that displacements can be measured using the DIC method with good precision.
- The comparative analysis of strain measurements using DIC and strain gauges across two axes (x and y) and two propeller models revealed important differences. While both methods detected similar strain patterns over time, DIC showed higher strain amplitudes and more pronounced peaks compared to strain gauges. Increasing the temporal filter may influence the results by smoothing the strain values. This indicates that filtering for dynamic measurements should be carefully adjusted during data post-processing to ensure accurate strain representation and avoid artificial distortions in the data.
- A Pearson correlation coefficient analysis was performed to statistically assess the agreement between DIC and strain gauge measurements. Strong correlation was observed in most cases (in the range of 0.79/0.94), particularly in the *y*-axis data, confirming the consistency of strain patterns. One outlier with low correlation was identified and attributed to local measurement artifacts.
- During the acceleration phase, when strain values were below 100 µm/m, increased fluctuations were observed in the DIC results. A comparison with strain gauge data indicates that small but real strain caused by inertial forces was present. Although these strain levels fall below the reliable detection threshold of the DIC system, they may still influence the measurement trend, leading to the observed fluctuations. Further investigation is needed to fully understand the interaction between low-magnitude dynamic strains and DIC measurement sensitivity.
- Noise analysis indicated significant discrepancies between the x and y directions in DIC strain measurements, with noise ranging from ±26 µm/m for the y direction and ±167 µm/m for the x direction. In contrast, strain gauge noise remained consistent and significantly lower, ranging accordingly from ±2.8 to ±6.9 µm/m.
- Plan for future work includes an investigation of the influence of spatial and temporal filtering parameters, as well as virtual strain gauge (VSG) size, on the noise and bias error of strain measurements. This error analysis will be continued in the next stage of the research, with a focus on quantifying their impact through controlled variation in filter settings and VSG sizes.

## Figures and Tables

**Table 1:** Measured tensile properties of the Complet LGF60-PA6 material.

Specimen Number	1	2	3	4	5
Tensile modulus [MPa]	10,532	10,691	10,259	10,794	10,558
Poisson’s ratio [%]	0.42	0.52	0.52	0.40	0.47
Tensile strength [MPa]	112.59	118.79	112.87	113.96	105.39

**Table 2:** Camera setup parameters.

	Left Image	Right Image
High-speed camera	Phantom VEO 410L	Phantom VEO710L
Lens	Nikkor 50 mm f/11	Nikkor 50 mm f/11
Sensor type	Mono CMOS Super 35	Mono CMOS Super 35
Sensors dimensions	25.6 × 16 mm	25.6 × 16 mm
Pixel size	20 µm	20 µm
Frames per second	5000 fps	5000 fps
Exposure time (global shutter)	10 µs	10 µs
Resolution set	1280 × 800	1280 × 800
Cameras stereo angle	18°	
Scale factor at propeller’s plane (measured on left camera)	≈0.2219 mm/px for 9 × 4.5E setup ≈0.2681 mm/px for 8 × 6SF setup	
Lighting	1× GsVitec Multiled MT—60,000 lumens Distance from the lamp to propeller 0.9 m	
Calibration panel	CP40-200 (GOM Correlate; Goslar, Germany)	
Calibration deviation	0.030/0.045 pixels	
Measuring volume	240/150/135 mm	

**Table 3:** Comparative analysis of strain measurements obtained with the DIC and strain gauges for Test Runs 1 and 2 of propellers APC 8 × 6SF and 9 × 4.5E.

Test Run	Parameter	DIC (εx)	Strain Gauge (εx)	DIC (εy)	Strain Gauge (εy)
TR1 8 × 6SF	Noise Level during Resting Phase [µm/m]	±167	±2.8	±26	±2.8
	Peak Strain (Max/Min) [µm/m]	427/−874	689/−350	1063/−564	643/−450
	Peak-to-Peak Strain of Impact [µm/m]	1301	1039	1627	1093
	Impact Peak Rise Time [ms]	3.7	2.8	2.1	1.7
	Correlation Coefficient [Time range: 36–67 ms]	0.94		0.84	
TR2 8 × 6SF	Noise Level during Resting Phase [µm/m]	±105	±2.8	±29	±2.8
	Peak Strain (Max/Min) [µm/m]	614/−938	736/−374	878/−440	653/−462
	Peak-to-Peak Strain of Impact [µm/m]	1552	1110	1318	1115
	Impact Peak Rise Time [ms]	Unclear	2.2	1.8	2.3
	Correlation Coefficient [Time range: 33–64 ms]	−0.16		0.85	
TR1 9 × 4.5E	Noise Level during Resting Phase [µm/m]	±174	±3.8	±69	±3.3
	Peak Strain (Max/Min) [µm/m]	884/−538	383/−371	654/−525	564/−500
	Peak-to-Peak Strain of Impact [µm/m]	1442	754	1179	1064
	Impact Peak Rise Time [ms]	2.1	2	2.1	2.2
	Correlation Coefficient [Time range: 10–24 ms]	0.79		0.89	
TR2 9 × 4.5E	Noise Level during Resting Phase [µm/m]	±162	±4.6	±33	±6.9
	Peak Strain (Max/Min) [µm/m]	504/−423	371/−369	802/−670	563/−500
	Peak-to-Peak Strain of Impact [µm/m]	927	740	1472	1063
	Impact Peak Rise Time [ms]	1.9	1.7	2.4	2.0
	Correlation Coefficient [Time Range: 10–24 ms]	0.90		0.90	

## Data Availability

The original contributions presented in this study are included in the article. Further inquiries can be directed to the corresponding author.
